# “Patient reported outcomes” following experimental surgery—using telemetry to assess movement in experimental ovine models

**DOI:** 10.1002/jor.23790

**Published:** 2017-12-05

**Authors:** Karin Newell, Jose Chitty, Frances M. Henson

**Affiliations:** ^1^ Department of Surgery University of Cambridge Cambridge United Kingdom; ^2^ Smartbell London United Kingdom; ^3^ Department of Veterinary Medicine University of Cambridge Cambridge United Kingdom

**Keywords:** telemetry, osteoarthritis, osteochondral, ovine, activity

## Abstract

Many potential treatments for orthopedic disease fail at the animal to human translational hurdle. One reason for this failure is that the majority of pre‐clinical outcome measurements emphasize structural changes, such as gross morphology and histology, and do not address pain or its alleviation, which is a key component of treatment success in man. With increasing emphasis on “patient reported outcome measurements (PROM)” in clinical practice, in this study we have used two different telemetric methods (geolocation and Fitbark activity trackers, Kansas City, MO) to measure movement behavior, i.e., an indirect PROM, in an ovine osteoarthritis induction and an osteochondral defect model performed in adult female Welsh Mountain sheep. This study demonstrates that both systems can be used to track movement and activity of experimental sheep before and after surgery and that the Geolocator system recorded a decrease in distance moved and activity at the end of the experimental period in both models. The Fitbark activity tracker also recorded significant alterations in movement behavior at the end of these studies and this method of recording showed a correlation between Fitbark data and radiography, macroscopic and histological scoring (well recognized outcome measurements), particularly in animals with large (10 mm) defects, i.e., more severe pathology. These results suggest that telemetry is able to track movement behavior in experimental sheep and that the methodology should be considered for inclusion in outcome measures in preclinical orthopedic research. © 2017 The Authors. *Journal of Orthopaedic Research®* Published by Wiley Periodicals, Inc. on behalf of Orthopaedic Research Society. J Orthop Res 36:1498–1507, 2018.

Orthopedic disease is an important economic and welfare issue affecting many millions of people. Large amounts of research time and money are spent globally on investigating the mechanisms underlying disease and on developing and evaluating effective therapies. Many different research strategies are employed to tackle key questions in orthopedic research, both in vitro and in vivo and the use of preclinical models in musculo‐skeletal research is widespread.^1^

Determining the outcome of these experiments used to evaluate the success or otherwise of medical device or biological therapy in orthopedic research is very well described in the literature.^1^, ^2^ Both in vivo (radiographic, MR imaging, and gait analysis^1^) and ex vivo parameters are routinely measured e.g., macroscopic scoring systems for quantifying the macroscopic appearance of the repair tissue^3^ and various histological‐histochemical grading systems.^4^, ^5^, ^6^, ^7^ However, despite the wide range of outcome measures, many drugs and devices that are judged to be successful in preclinical trials fail at the point of translation from animal studies to human studies, for a variety of reasons.^8^, ^9^ One of these reasons may be that traditional outcome measures assume that structural changes in tissues away from a normal state represent a negative outcome of treatment and do not consider the pain arising from the orthopedic treatment or subsequent interventions.

Measuring pain in animals, is difficult,^10^, ^11^ particularly herd animals such as sheep. In orthopedic research, pre‐clinical studies indirectly address pain by quantifying clinical lameness and weight bearing on the affected limb,^12^, ^13^ while in clinical human patients increasing emphasis is being placed on “patient reported outcome measures” (PROM) to judge success of intervention and therapy.^14^ Clearly direct PROM are not possible in animals, however, with the advent of telemetry technology, new opportunities arise to remotely monitor the movement behavior of animals used in preclinical orthopedic experiments, potentially establishing novel methods of drug and device evaluation.

The sheep is a commonly used translational model for evaluating efficacy of potential treatments for orthopedic research^15^, ^16^, ^17^ and is a species in which it is difficult to assess functional outcomes in a flock situation. The objective of this study was to identify whether telemetry could be used to monitor movement behavior in experimental sheep. The specific aims of the study were to i) describe the use of two different commercially available telemetric systems to measure movement behavior in experimental sheep before and after surgery and ii) to evaluate correlations between currently used structural outcome measurements and telemetry in these systems.

## METHOD

This study received approval from both the Animal Welfare and Ethical Review Body, University of Cambridge and the UK Home Office Project Licence number 70–7740.

### Animals

Forty skeletally mature Welsh Mountain Sheep (mean age 3.5 ± 0.6 years) were included in the study.

### Telemetric Systems

Two different commercially available telemetric systems were used to record animal activity; “Fitbark” activity trackers (FATs) (Fitbark) and a Cluster Geo‐locator system (Omnisense, Cambridge, UK).

## Study Design

### Study 1 Osteoarthritis (OA) Induction Model

Transection of the medial mensiscus was performed to induce OA over 12 weeks^18^ in 28 animals. The movement behavior of all 28 were tracked with the Omnisense system. A subset of 12 sheep chosen at random by a remote investigator, were also fitted with an individual FAT.

### Study 2 Osteochondral Model

Twelve animals were used to investigate the efficacy of using FATs following the creation of 7 mm (*n* = 6) and 10 mm (*n* = 6) osteochondral defects of the medial femoral condyle (MFC) over 26 weeks.

## Animal Anesthesia, Preparation, and Surgical Techniques

General anesthesia was induced with an injection of thiopentone (3 mg/kg) and maintenance achieved via inhalational anesthetic. Perioperative analgesia was provided by pre‐operative intramuscular 4 mg/kg carprofen. Antibiotic prophylaxis was given via intramuscular 10 mg/kg procaine penicillin. The basic surgical procedure was identical for all subjects and performed under strict asepsis. Each stifle was physically examined for any abnormalities while anesthetized. If any gross instability or pathology was found, the animal was excluded from participation within the study.

For osteochondral defects, the animal was placed in a dorsal recumbency position and, following surgical preparation, the left stifle joint opened via a medial parapatellar approach.^19^ Following patella subluxation and elevation of the fat pad to allow access to the condyle, a full thickness 7 mm diameter ×6 mm deep or 10 mm diameter ×8 mm deep osteochondral defect was created in the MFC using a hand drill. Acute haemostasis within the defect was achieved using pressure from a surgical swab. Defects were positioned in the center of the condyle, 10 mm distal to the condyle groove junction and aligned with the medial crest of the trochlea groove.

For meniscal defects, the animal was placed in dorsal recumbancy and the medial femoro‐tibial joint opened from the medial aspect, cranial to the collateral ligament and leaving the medial collateral ligament intact. The superior surface of the medial meniscus was identified and carefully transected as described previously.^18^ In both surgical procedures the joint was closed in a standard fashion; vertical matress sutures were used to close the incision through the joint capsule (0 vicryl, Ethicon) and the skin closed with vicryl (3–0, Ethicon) (absorbable suture used to avoid suture removal post‐operatively). No splints, casts or immobilisation techniques were used in any animal. Post‐operative analgesia was provided for the first 72 h following surgery (intramuscular 4 mg/kg carprofen once a day).

Postoperatively, animals were allowed to fully weight bear, but kept in small pens for 48 h to reduce ambulation. All animals were then maintained for a further 5 days in a large indoor pen before being kept outdoors in the field fitted with tracking sensors (see section below). Animals were killed with an overdose of injectable anesthetic at 26 weeks post surgery (ostechondral defects) and 12 weeks (meniscal defects).

## Use of Cluster Geolocator System

The OA induction model sheep were fitted with mobile location sensors (GCN‐533, Omnisense) by securely fixing the sensor to a ram harness worn by the sheep. Sensors were placed on the dorsum of the sheep. Batteries were replaced when required as indicated by the software programme. The field in which the sheep were kept was equipped with 12 fixed sensors (GCN‐533), including one fitted as a “Gateway” sensor. Data were recorded from each individual animal and collected via a “whereBox Cluster Location System” (Omnisense) using “omniWhere and Location Engine Software” (Omnisense). The geolocation system provides three‐dimensional position value and acceleration data from which distance and activity levels were calculated.

The frequency of data transmission was set at 0.3 Hz. Each position was provided with a confidence value (from 0 to 1) and the system was calibrated by moving the sensors through a series of known patterns as well as looking at sensor performance in fixed positions. Data was gathered using an A2E™ phone application (Smartbell, Cambridge, UK) and processed using A2E™ software. Calibration and testing of different configurations were performed and a minimum confidence value of 0.9 was selected with an activity threshold filter of 1.2 G to reduce positional noise when the sheep were inactive. With these settings, distance travelled was within 10% of actual values.

Distance travelled and activity (whether moving or stationary) was analyzed for each individual animal and scaled to the readings obtained prior to surgery so that comparisons between animals could be made. Data were analyzed from Saturdays and Sundays over the period of the experiment. These days were chosen as there was minimal disruption to the animals due to on‐farm activity. Data were collected for three periods during the experiment—2 weeks prior to surgery (weeks −2 to 0 of the experimental period), the 2 weeks following post surgery (weeks 1–2 where “week 0” is the week immediately following surgery) and the final 2 weeks of life at the end of the experiment (weeks 11–12).

## Activity Tracker Data Recording

Twelve osteochondral defect model sheep and 12 OA induction model sheep were fitted with FATs attached to a collar. FATs were removed once a week and the battery charged. Individual animal data was uploaded to the “Fitbark” central server automatically. Activity levels were then downloaded onto a mobile telephone through the “Fitbark” app. The “Fitbark” app divides the periods of activity during the day into three; “play,” “active,” and “rest,” corresponding to high activity, medium activity, and no movement (standing still or sleep).

“Play,” “activity,” and “rest” was analyzed for each individual animal. Data were analyzed from Saturdays and Sundays over the period of the experiment. Data were collected for three periods during the experiment −2 weeks prior to surgery, the 2 weeks immediately post surgery and the final 2 weeks of life at the end of the experiment for OA induction model and for the final 2 weeks of life for the osteochondral defect model.

## Radiographic Evaluation

Following euthanasia cranio‐caudal radiographs were obtained of the operated limbs. The radiographs were scored using the Kellgren‐Lawrence scoring system by blinded observers (Table 1). A high Kellgren‐Lawrence score indicates increased damage to the joint.

**Table 1:** The Kellgren–Lawrence Scoring System

Score	Description
1	No change
2	Doubtful narrowing of the joint space and possible osteophyte lipping
3	Definite osteophytes and possible narrowing of the joint space
4	Large osteophytes, marked narrowing of joint spaces, severe sclerosis, and definite deformity of bone contour

## Postmortem Evaluation—OA Induction Model

The gross morphology of the operated joint was scored blindly at post mortem using a scoring system described by Delling et al.^20^ Each joint was divided into four anatomical regions: medial femoral condyle, lateral femoral condyle, medial tibial plateau, and lateral tibial plateau. At each anatomical site a surface damage score (total possible 16) (Table 2A) and an osteophtye score (total possible 12) (Table 2B) was given. A high gross morphology score indicates increased damage to the joint.

**Table 2:** Post‐Mortem Scoring for Osteoarthritis Induction Model

Score	Description
A: Surface damage score	
0	Normal
1	Surface roughening
2	Fibrillation and fissures
3	Small erosions down to subchondral bones (<5 mm diameter)
4	Larger erosions down to subchondral bone (>5 mm diameter)
B: Osteophyte score	
0	Normal
1	Mild osteophyte development (<2 mm outgrowth or <20% of join
2	Moderate osteophyte development (2–4 mm outgrowth or 20–50% of joint margin)
3	Large osteophyte development (>4 mm outgrowth or >50% of joint

## Postmortem Evaluation—OC Model

At post mortem joints were opened and the surface of the osteochondral defect sites blindly scored using the International Cartilage Repair Society macroscopic scoring system (Table 3).^21^ In contrast to the post mortem evaluation system used to score the OA induction model, a high gross morphology score in the OC model indicates decreased damage to the joint.

**Table 3:** Post‐Mortem Scoring for Osteochondral Model

Description	Description	
Degree of defect repair	In level with surrounding cartilage	4
	75% repair of defect depth	3
	50% repair of defect depth	2
	25% repair of defect depth	1
	0% repair of defect depth	0
Integration to border zone	Complete integration with surrounding cartilage	4
	Demarcating border <1 mm	3
	3/4 of graft incorporated, ¼ with notable border >1 mm wide	2
	1/2 of graft incorporated, ½ with notable border >1 mm wide	1
	From no contact to ¼ of graft integrated	0
Macroscopic appearance	Intact smooth surface	4
	Fibrillated surface	3
	Small, scattered fissures or cracks	2
	Several, small or few but large cracks	1

## Histological Evaluation—OA Induction Model

Osteochondral samples (6 × 6 mm) were collected from the load‐bearing cartilage regions of the lateral and medial femoral condyles and lateral and medial proximal tibial condyles. Each sample was obtained from the central portion of the joint, determined using measurement of each joint. Samples were decalcified in formic acid/sodium citrate over 2 weeks and paraffin‐embedded sections (10 μm thickness) prepared. Sections were stained with Safranin O‐Fast Green. Sections were obtained from the four compartments of the operated joint and blindly scored by one investigator using a modified Mankin score, the scores of all four compartments were added to give a final Mankin score (Table 4). A high Mankin score indicates increased damage to the joint.

**Table 4:** Modified Mankin Score

Description	Description	
Degree of defect repair	In level with surrounding cartilage	4
	75% repair of defect depth	3
	50% repair of defect depth	2
	25% repair of defect depth	1
	0% repair of defect depth	0
Integration to border zone	Complete integration with surrounding cartilage	4
	Demarcating border <1 mm	3
	3/4 of graft incorporated, ¼ with notable border >1 mm wide	2
	1/2 of graft incorporated, ½ with notable border >1 mm wide	1
	From no contact to ¼ of graft integrated	0
Macroscopic appearance	Intact smooth surface	4
	Fibrillated surface	3
	Small, scattered fissures or cracks	2
	Several, small or few but large cracks	1

## Histological Evaluation—OC Model

The OC defect site was collected and specimens were decalcified in formic acid/sodium citrate over 4 weeks, prior to routine paraffin processing. Sections of 10 μm thickness were made through the central portion of the defect. Sections were stained with Safranin O/Fast Green. Sections were blindly scored by one investigator, using a modified O'Driscoll score (Table 5). A high modified O'Driscoll score indicates damage to the joint.

**Table 5:** Modified O'Driscoll Score

Description	Description	
Degree of defect repair	In level with surrounding cartilage	4
	75% repair of defect depth	3
	50% repair of defect depth	2
	25% repair of defect depth	1
	0% repair of defect depth	0
Integration to border zone	Complete integration with surrounding cartilage	4
	Demarcating border <1 mm	3
	3/4 of graft incorporated, ¼ with notable border >1 mm wide	2
	1/2 of graft incorporated, ½ with notable border >1 mm wide	1
	From no contact to ¼ of graft integrated	0
Macroscopic appearance	Intact smooth surface	4
	Fibrillated surface	3
	Small, scattered fissures or cracks	2
	Several, small or few but large cracks	1

## Statistical Evaluation

Correlations between telemetry system data and animal data were made by deriving the Pearson's correlation coefficient for each data set. Correlations were considered to be significant if *r* > 0.5. Oneway ANOVA and Tukey's multiple comparison test was used to identify differences between groups.

## RESULTS

### Cluster Geolocator System

Fixing individual mobile sensor locators to the sheep via a ram harness was well tolerated by the animals and did not lead to any complications or issues.

The Cluster Geolocator system was used for 14 weeks, during the experimental period the batteries on the sensors attached to the animals were changed approximately every 10 days. The system of data recording was not entirely stable through the experimental period with approximately 80% of desired data recorded. The Omnisense geolocator system successfully recorded the distance moved (measured in *m*, with a 10% accuracy as described above) and the activity levels of the sheep (designated as “units of activity”).

### Activity Tracker Use

Fixing the FAT to the individual sheep collar was well tolerated by the animals and did not lead to any complications or issues. The use of the FAT allowed recordings of animal activity to be made and retrieved via a mobile telephone. The system of recording was robust and reliable with no data drop‐out. The FAT was successfully able to categorise different periods of sheep activity into “play,” “active,” and “rest” which were expressed as minutes per day.

### OA Induction Model Telemetry Results

#### Cluster Geolocator Results—Distance

**Figure 1:**
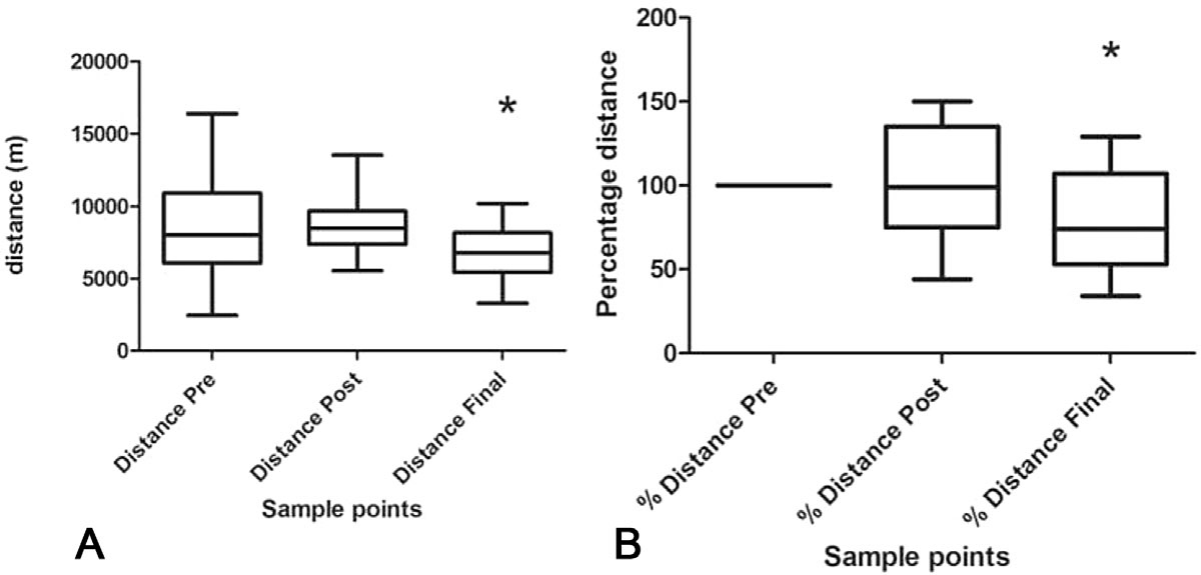
Distances travelled by sheep before surgery, 1–2 weeks post surgery and in the final 2 weeks of life post surgery in an OA induction model in 28 sheep. (A) Absolute distances travelled. There was a significant reduction in the final 2 weeks compared to pre‐ and immediately post‐surgery (*). (B) Individual percentage distance travelled by sheep. There was a significant reduction in distance travelled in the final 2 weeks compared to pre‐ and immediately post‐surgery (*).

Cluster Geolocator recordings of 28 OA induction model sheep recorded that the mean distance travelled prior to surgery as 8363 ± 3126 m, 8687 m ± 1996 after surgery and dropping to 6786 m ± 1960 at the end of the experimental period (all data expressed per 24 h). This represented a significant decrease in distance travelled at the end of the experiment compared to prior to and after surgery (*p* < 0.0001) (Fig. 1A). These figures, when expressed for each individual animal to reduce inter‐animal variation, represent distance travelled of 100% pre surgery, 102% post surgery, and 80% at the end of the experimental period (Fig. 1B). These represented a significant decrease in relative distance travelled by individual animals at the end of the experiment compared to prior to and after surgery (*p* < 0.002).

#### Cluster Geolocator Data—Activity

**Figure 2:**
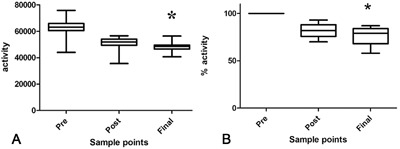
“Activity” of sheep before surgery, 1–2 weeks post surgery and in the final 2 weeks of life post surgery in an OA induction model in 28 sheep. (A) Absolute activity. There was a significant reduction in the final 2 weeks compared to pre‐ and immediately post‐surgery (*). (B) Percentage ‘activity’ of sheep. There was a significant reduction in activity in the final 2 weeks compared to pre‐ and immediately post‐surgery (*).

Cluster Geolocator recordings of 28 OA induction model sheep recorded that, with this telemetry system, the mean activity prior to surgery was 62527 ± 6773 per 24 h, 50381 ± 5112 per 24 h after surgery and dropping to 48566 ± 3206 at the end of the experimental period (Fig. 2A). These figures, when scaled for each individual, represent activity of 100% pre surgery, 81.5 ± 11.9 % post surgery and 79 ± 12% at the end of the experimental period (Fig. 2B. There was a statistically significant difference in the activity of the sheep at the end of the experimental period compared to before surgery, both in absolute activity and relative activity (*p *< 0.05).

### FAT Tracker Results

**Figure 3:**
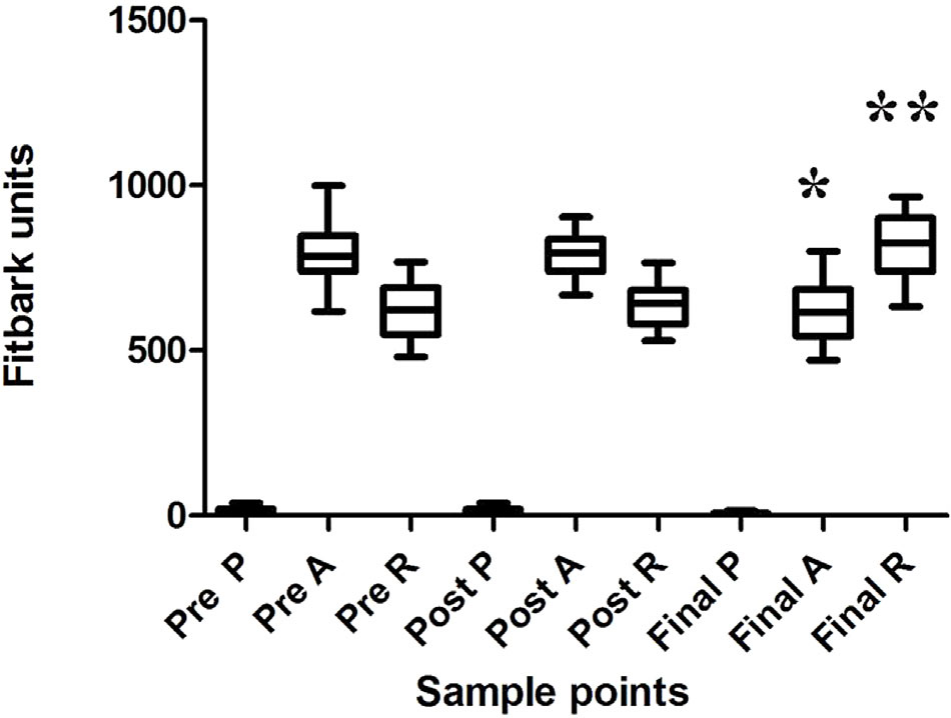
Fitbark activity tracker (FAT) data of sheep before surgery, 1–2 weeks post surgery and in the final 2 weeks of life post surgery in an OA induction model in 12 sheep. There was a significant reduction in “activity” (*) and a significant increase in rest in the final 2 weeks of life (**) compared to pre‐ and post‐surgery.

FAT data of 12 OA induction model sheep recorded that the mean play was 15.1 ± 7.2 min/day prior to surgery, 13.8 ± 8.8 min/day post surgery and 6.9 ± 2.8 min/day at the end of the experimental period. The mean activity was prior to surgery was 810 ± 204 min/day prior to surgery, 788.5 ± 57.8 min/day post surgery and 620 .1 ± 97 min/day at the end of the experimental period and the mean rest was 630 ± 76 min/day prior to surgery, 640 ± 54 min/day post surgery and 816.1 ± 103.3 min/day at the end of the experimental period (Fig. 3). There was a statistically significant difference in the activity and rest of the sheep at the end of the experimental period compared to before and post surgery (*p *< 0.05); there was less activity and more rest at the end of the experimental period.

### Osteochondral Defect Model Telemetry Results

#### FAT Data

Following from the recording of activity in the OA induction model a second cohort of animals was recruited into the study, an osteochondral defect model. This second cohort of animals had 7 and 10 mm osteochondral defects made in their medial femoral condyles.

**Figure 4:**
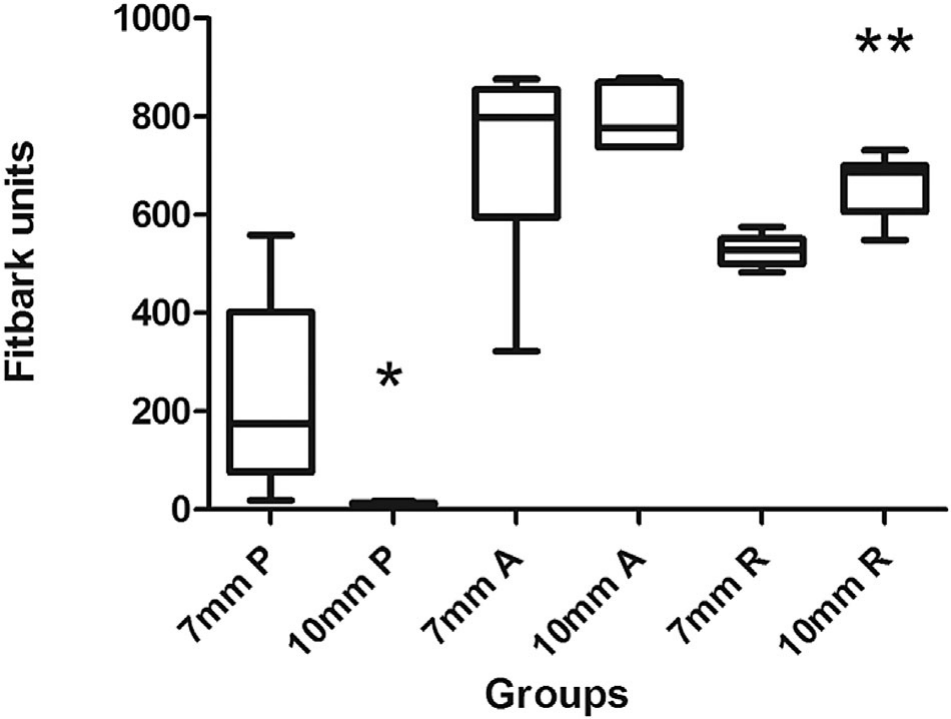
Fitbark activity tracker (FAT) data from sheep that had undergone osteochondral defect surgery. Data acquired from sheep in the final 2 weeks of life (weeks 25 and 26) post surgery. There was a significant reduction in “play” in animals with 10 mm osteochondral defects compared to animals with 7 mm osteochondral defects and a significant increase in “rest” in animals with 10 mm osteochondral defects compared to animals with 7 mm osteochondral defects.

FAT data of 12 osteochondral defect model sheep recorded that the mean time spent in “play” was 13 ± 1.7 min/day, “activity” 794 ± 150 min/day, and “rest” 594 ± 85 min/day. When evaluating 10 mm defect animals compared to 7 mm defect animals, there was a significant difference in the amount of time spent in “play” (2 ± 2 min/day for 10 mm defect animals compared to 15 ± 1.1 min/day for 7 mm defect animals, *p* = 0.02) and “rest” (661 ± 64 min/day for 10 mm defect animals compared to 527 ± 31 min/day for 7 mm defect animals, *p* = 0.01), indicating that animals with 10 mm osteochondral defects played for significantly less time and rested for significantly more time than animals with 7 mm defects at the same site (Fig. 4).

#### Correlation of Telemetry Data With “Standard” Outcome Measures

In order to contextualize the telemetry data, the results from both recording systems were correlated with Kellgren‐Lawrence scores of the joints at the end of the experimental period, the appropriate gross morphology score of the joint at post mortem and the appropriate histological score of the tissue (Tables 6 and 7).

**Table 6:** Correlation of Distance (*D*), Percentage Distance, Activity (*A*), Percentage Activity With Lawrence–Kellgren Radiographic Score, Macroscopic Score, and Histological Score in OA Induction Model at 12 Weeks Post Surgery Using a Geolocator Telemetry System

Parameter	*D*	%*D*	*A*	%*A*
Lawrence–Kellgren score	0.15	0.42	0.18	0.33
Macroscopic score	−0.06	0.365	0	0.34
Histological score	−0.18	0.29	−0.18	0.26

**Table 7:** Correlation of Play (*P*), Activity (*A*), and Rest (*R*) With Lawrence–Kellgren Radiographic Score, Macroscopic Score, and Histological Score in OA Induction at 12 Weeks Post Surgery and in and OC Model 26 Weeks Post Surgery Using a Fitbit Telemetry System

	OA Model *P*	OA Model *A*	OA Model *R*	OC Model *P*	OC Model *A*	OC Model *R*	OC Model 10 mm *P*	OC Model 10 mm *A*	OC Model 10 mm *R*
Lawrence–Kellgren score	0.3	0.23	−0.3	−0.41	−0.3	0.6	−0.1	−**0.94**	**0.65**
Macroscopic score	−0.13	−0.19	0.1	0.25	−0.13	0.1	0.65	0.12	0.26
Histological score	−**0.54**	−0.43	0.42	−0.23	−0.14	0.36	0.46	−**0.74**	**0.78**

Numbers highlighted in bold signify a Pearson coefficient of > 0.5.

#### Correlation of Telemetry Data With Kellgren–Lawrence (K–L) Score

The K‐L score was used to quantify radiographic changes within the operated limb with a high K–L score indicates an increase in damage in the joint.

In the OA induction model, using the Geolocator telemetry system, there were no significant correlations between the absolute and percentage distances and activity of the sheep. Similarly, the analysis of a 12/28 animals from the same group using a Fitbark system also failed to show significant correlations. In the OC model, using the Fitbark system, there was a positive correlation between the amount of rest and the radiographic score when considering the animals as a group and in the 10 mm defect animals alone (*r* = 0.6 and 0.65, respectively). In addition, there was a very strong correlation between activity and radiographic score in the 10 mm defect animals alone (*r* = −0.94), compared to the OC model as a group (*r* = −0.3).

#### Correlation of Telemetry Data With Post Mortem Macroscopic Score

Post mortem macroscopic scoring systems were used to quantify gross damage to the joints after surgery. For the OA induction model a high score indicated increase damage to the joint, for the OC model a high score indicated reduced damage to the joint.

In the OA induction model, neither measurements using the Geolocator telemetry nor the Fitbark system showed significant correlations between the absolute and percentage distances and movement behavior. In the OC model, when the animals were considered as a group, there were no positive correlations between macroscopic damage and play, activity or rest. However, in the 10 mm defect animals there was a positive correlation between play and macroscopic score (*r* = 0.65).

#### Correlation of Telemetry Sata With Histological Score

Histological scoring systems were used to quantify microscopic damage/repair in the joints after surgery. For both models, high scores indicated increased damage to the joint.

In the OA induction model, using the Geolocator telemetry system, there were, again, no significant correlations between the absolute and percentage distances and activity of the sheep. Analysis the Fitbark system showed that there was a significant correlation between play and histological score (*r* = −0.54) and high, but not significant *r* values for activity (−0.43) and rest (0.42). In the OC model group there were no significant correlations in the group when considered as a whole, however in the 10 mm defect animals there were strong correlations between histological score and activity (*r* = −0.74) and rest (*r* = −0.78).

## DISCUSSION

In this study we have demonstrated the use of two commercially available telemetric systems for monitoring the activity of sheep before and after experimental orthopedic surgery and shown that each system can be used to monitor free movement of animals. These findings have significant implications for the use of large animals in experimental orthopedic surgery in two key areas; animal welfare and the quantification of “patient reported data,” a hitherto unachievable goal.

Telemetric monitoring of movement of animals has been widely used to tracking the movement of wild animals, particularly aquatic ones such as fish.^22^ Telemetry systems have also been used to monitor the behavior and physiology of farm animals, particularly dairy cows.^23^, ^24^ Telemetry in experimental animals has been reported but is primarily confined to recording metabolic data, such as blood pressure and heart rate,^25^ and in recording more complex physiological processes such as EEG recording.^26^ The authors are unaware of any previous published data on the use of telemetry to record movement in experimental large animals.

Telemetric monitoring of movement behavior in experimental animals offers potentially significant benefits in the assessment of pain and behavior, primarily because the animals’ behavior is monitored remotely with no interference from the presence of humans, but also because it provides objective, quantitative data on movement rather than subjective assessment of movement behavior. Assessment of pain in animals is difficult, particularly in the absence of agreed pain criteria^27^ and for orthopedic pre clinical reporting, has relied primarily on clinical lameness and weight bearing on the operated limb. Measuring both of these parameters only provides data for the few minutes of recording/obervation over a 24 h period and both of which require an active human presence, which could alter the data collected.^28^ In contrast, telemetry measurements are constant throughout the 24 h period, do not require human contact and mat, therefore, more accurately represent animal patient derived movement outcomes. Telemetry can therefore be useful in the evaluation of orthopedic treatments and in the evaluation of animal welfare, as cut off points such as “an increase in rest time of greater than 50% compared to pre surgery levels” could be potentially used as experimental end points.

Both the Omnisense Geolocator system and the Fitbark system were used to record animal movement during a study to induce OA following medial meniscal transection. This surgical technique is a progressive disease model that would be expected to cause reduced mobility after time and that has been shown to cause reduced weight bearing and gross and histopatholgoical changes consistent with OA.^18^ Measurements of movement in this model, using the Geolocator, did not find a significant difference between pre and post surgery distance moved and “activity,” but distance and “activity” were significantly reduced at the end of the 12 week experimental period. Similarly, when using a Fitbark to measure movement data, this recording system showed no significant difference between pre‐ and post‐surgery movement levels, but a significant decrease in “activity” and increase in “rest” at the end of the experimental period. One limitation of this study is that it only includes experimental subjects, i.e., there are no normal sheep or sham‐operated controls included to mitigate against confounding factors such as effect of surgery, season, weather, familiarity with surroundings. This omission may influence the interpretation of these results, however the data presented in this study does demonstrate that the technology can provide on functional behavior before and after experimental surgery in sheep and current studies are on‐going to include cohorts of control animals. Both of these two telemetric methods can be used to monitor movement behavior during ovine OA induction models, however, the cost and ease of use of the Fitbark system, suggested to the authors that this system could be more likely to be widely adopted for such measurements.

In order to investigate the ability of the Fitbark system to distinguish differences in movement behavior between animals with intra‐experimental differences, a further cohort of 12 animals that had undergone osteochondral defect surgery were evaluated over 2 weeks at the end of a 6 month experimental period. In this group six animals had a 7 mm defect of the medial femoral condyle (moderate defect that would be expected to heal without intervention^29^), the other 6, a 10 mm defect of the medial femoral condyle, which represents a large defect that would lead to joint collapse, i.e., a lesion that would be predicted to be more severe, cause more pain and impact more on movement. Using Fitbark monitoring there was a significant decrease in “play” and a significant increase in “rest” at the end of the experimental period in the 10 mm defect animals compared to the 7 mm defect animals, indicating that using Fitbark to record movement data could provide information within a similar experiment and confirming the 10 mm defect as causing more impact on movement than a smaller osteochondral defect.

One current limitation of the technologies used is that the categories of movement behavior, i.e., “activity,” “rest,” “play” are set internally within each telemetric system by the service provider and that we do not yet have detailed information on what exactly these broad categories fully represent. Work is currently on‐going to provide further detail on the absolute meaning of these categories, which will hopefully refine further the use of the telemetric systems in pre clinical orthopedic research.

While the use of telemetry to record movement data during orthopedic experimental procedures is interesting, for it to become an acceptable method of experimental evaluation, it is important to evaluate how the data acquired relates to currently accepted outcome measurements. However, in the evaluation of any correlations, it must be noted that that movement behavior of the animals may be more likely to represent pain or discomfort in the animals (and may be more relevant in the development of potential therapies), while other, more traditional, outcomes are primarily measuring structural changes, either gross or microscopic within the joint. Therefore, while correlations between telemetric data and more traditional outcome measurements would be satisfactory, lack of correlation may suggest fundamental differences in the data acquired and what it may mean.

Three outcome measurements were used to identify correlations with telemetry; radiographic changes, gross morphology scoring, and histological scoring. In the OA induction model, while both the Omnisense Geolocator system and the FAT showed significant alterations in movement at the end of the experimental period consistent with the degenerative nature of the model, no significant correlation could be found between telemetric outputs and radiographic changes or gross morphology, although there were correlations with histological morphology. In contrast, in the osteochondral defect model, the FAT did show correlations with more of these standard outputs, particularly with radiography. Of note, the correlations were strongest in the 10 mm defect group, i.e., those animals with most structural damage (and, by assumption, pain). This finding may suggest that the geolocator technology is more suited to more severe disease phenotypes and that the technology used in this study is not currently suitable for distinguishing between subtle differences in joint disease. Indeed, within the Omnisense system, there was a known 10% level of inaccuracy in the data acquired, reflecting the inadequacy within this system. This result may also indicate that subtle differences in joint disease within an experiment are not clinically significant, i.e., they do not cause sufficient pain to modify movement behavior, or, this result may be suggesting that traditional outcome measurements are not the most satisfactory way to evaluate the effect of disease models. Clearly more work is required in this area and experiments are currently underway to progress our knowledge of the interplay between movement behavior, pain and outcome measures in experimental ovine models.

In conclusion, this study has reported, for the first time, the use of telemetry in experimental orthopedic models and demonstrated that movement behavior changes post operatively in both OA induction and osteochondral defect models. This study also shows some correlation with accepted outcome measures, particularly in the most extreme disease phenotype studies, and suggests that telemetric monitoring of experimental large animals may be a useful functional output in orthopedic research.
